# Analysis of the Effect of Component Ratio Imbalances on Selected Mechanical Properties of Seasoned, Medium Viscosity Bone Cements

**DOI:** 10.3390/ma15165577

**Published:** 2022-08-13

**Authors:** Jakub Szabelski, Robert Karpiński, Przemysław Krakowski, Mariusz Jojczuk, Józef Jonak, Adam Nogalski

**Affiliations:** 1 Department of Computerization and Production Robotization, Faculty of Mechanical Engineering, Lublin University of Technology, Nadbystrzycka 36, 20-618 Lublin, Poland; 2 Department of Machine Design and Mechatronics, Faculty of Mechanical Engineering, Lublin University of Technology, Nadbystrzycka 36, 20-618 Lublin, Poland; 3 Chair and Department of Trauma Surgery and Emergency Medicine, Medical University of Lublin, Staszica 11, 20-081 Lublin, Poland; 4 Orthopaedic Department, Łęczna Hospital, Krasnystawska 52, 21-010 Leczna, Poland

**Keywords:** bone cement, mechanical parameters, compressive strength, component ratio inaccuracy, Ringer solution, seasoning, degradation

## Abstract

The paper presents the results of experimental strength tests of specimens made of two commercially available bone cements subjected to compression, that is a typical variant of load of this material during use in the human body, after it has been used for implantation of prostheses or supplementation of bone defects. One of the factors analysed in detail was the duration of cement seasoning in Ringer’s solution that simulates the aggressive environment of the human body and material degradation caused by it. The study also focused on the parameters of quantitative deviation from the recommended proportions of liquid (MMA monomer, accelerator and stabiliser) and powder (PMMA prepolymer and initiator) components, i.e., unintentional inaccuracy of component proportioning at the stage of cement mass preparation. Statistical analysis has shown the influence of these factors on the decrease in compressive strength of the cements studied, which may be of significant importance in operational practice.

## 1. Introduction

Bone cements are widely used in orthopaedic surgery. Most frequently, they are used as a binding material between joint prostheses and bone in cases of total joint arthroplasty (TJA). They are also used as a filling material in cases of bone loss in the course of replacement surgery after bone tumour resection and after trauma. On annual basis, there is a rise in the number of joint replacement surgeries performed all over the world. Between 2003 and 2014 the increase in the number of TJA performed reached 115% [1]. Moreover, it is estimated that the rise in the number of TJA will increase continuously and by 2040 the increase will reach 400% [2]. Several factors are responsible for this phenomenon including longer life expectancy, which with more than 50% probability by 2030 will break the 90 year barrier [3]. Osteoarthritis (OA), which is the main indication for TJA is considered as a disease of elderly population as over one-third of the population over 65 years old present OA in at least one joint [4,5,6,7]. However, other factors such as obesity, sports, or socio-economic status can trigger development of OA in younger population [8,9,10]. Even with appropriate conservative treatment, OA will progress over time and eventually require surgical treatment [11]. Multiple methods of minimally invasive treatment were developed over the period of time [12,13,14], nevertheless, for end-stage disease TJA is a gold standard treatment. Properly performed TJA restore painless movement of the affected joint; however, TJA has its limits in regard to prostheses survival time [15]. The most common reasons for revision of TJA are instability, aseptic loosening and infection [16]. In cemented TJA, the only binding between bone and endoprosthesis is bone cement introduced by Charnley in 1950 [17], therefore disturbances in its mechanical properties are an important factor in TJA survival rate. As shown by other researchers, cemented TJA have higher load-to-failure than cementless versions of TJA [18]. Nevertheless, endoprosthesis as well as bone cements are subjected to highly aggressive environment in human body, and also have to withstand high loads during activities of daily living (ADL), reaching 300–400% of body weight during walking [19]. Techniques of cement application has evolved since introduced by Charnley from digital application through syringe application and vacuum mixing and delivery to pressurisation [20] in order to create best and most resilient binding between bone and prosthesis. Proper cementation technique is prerequisite for best mechanical properties, as it was shown that, bone cement preparation protocol and deposition of prosthesis components play an important role in the process of aseptic loosening of TJA [21,22]. Despite multiple studies there is no consensus on exact explanation of aseptic loosening in cemented TJA. Factors such as residual air bubbles [23], debonding [24], cement fractures [24], or wear products produced from bone cement [25] have been proposed in the literature as potential factors decreasing bone cements durability. Each manufacturer provides surgeons with strict cement preparing instructions. Nevertheless, on multiple occasions, orthopaedic surgeons do not follow appropriate cementation techniques [26]. As shown by a national survey among British orthopaedic surgeons, over 50% do not use pulsative lavage, and more than 10% do not dry the bone before cementing the implants [27]. Such approach can multiply detrimental effects, which human body has on bone cements for example by absorbing water and other bodily fluids, which will change its properties [28].

The bone—bone-cement connection is known as one of the weak zones of the prosthesis-cement-bone system because it does not adhere to the bone [29]. Many external and internal factors influence the mechanical properties of bone cement. External factors include the speed and time of mixing and the mixing method used [30,31,32], the precooling of the monomer and the degree of porosity [29]. Mixing can be done manually, using centrifugation or vacuum technology [20]. Internal factors that can influence the mechanical properties include: monomer and powder composition, powder particle size, shape and size distribution and powder to liquid ratio [33,34], accidentally introduced contaminants naturally occurring in the surgical field such as physiological fluids, blood and bone fragments [35,36,37,38,39,40] and intentional admixtures to improve the properties of the resulting bone cement. Intensive research to obtain bone cements with improved mechanical, thermal and biological properties is currently being conducted. These mainly involve the introduction of small amounts of components such as carbon [41,42], zirconium [43,44], titanium [45,46,47] or graphite fibres [48], graphene oxide [49,50,51], bioactive glasses [52], nanosilver [52,53] into the bone cement mass at the preparation stage. Admixtures such as polydioxanone (PDO) [52], cellulose [54,55], mesoporous silica nanoparticles [56,57], aramid [58,59], tricalcium phosphate (TCP) [60] or hydroxyapatite (HA) [61,62,63,64,65] and various antibiotics such as gentamicin, tobramycin, erythromycin, cefuroxime, vancomycin or colistin [66,67,68] are also introduced.

In surgical practice, at the stage of preparing two-component bone cements, minimal operator error is possible in terms of disturbing the correct proportion of the components of the cement being prepared. This can lead to changes in the strength parameters of the finished cement and, if significantly worsened, can also lead to damage to the human body. Ultimately, it may result in deterioration of the parameters of the bone-cement-prosthesis interface, leading to loosening of the prosthesis and, in the worst case, even requires revision surgery [34].

Considering the fact that bone cement is the weakest link of the bone-cement-prosthesis system, it seems extremely important to determine the influence of factors that may cause a premature loss of mechanical properties and as a result lead to prosthesis loosening. The purpose of this study was to estimate the effect of the disruption of the ratio of bone cement components bound on selected mechanical properties of typical commercially available PMMA-based bone cements and to estimate the deviation limits to maintain the mechanical parameters at which the material can be implanted.

## 2. Materials and Methods

**Figure 1:**
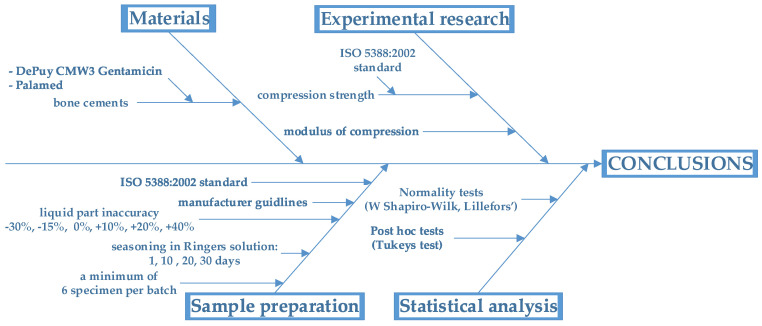
Research plan and main objectives of the analysis of results.

Because of the nature of the material’s function after implantation (bone cement in joint endoprostheses is mainly subjected to compressive loading), it was decided that the best method to assess the effect of component disruption and seasoning in Ringer’s solution would be physical tests involving axial compression of cylindrical cement samples. Figure 1 illustrates the test methodology, and a detailed description of the individual stages is presented in the following chapters.

### 2.1. Materials and Sample Preparation

The study analysed standardised samples made from two commercially available cements: De Puy CMW 3 Gentamicin and Palamed. According to the standard (ISO 5833:2002 (E) [69]—Implants for surgery—Acrylic resin cements, Annex E—Determination of compressive strength of polymerised cement) cylindrical specimens of ø12 × 6 mm (±0.1 mm) were made. The specimens were prepared in the form of a variant complying with the manufacturer’s recommended proportion of components and with disruptions to this proportion in order to estimate the potential effect of these distortions on the mechanical parameters of the final material. Disturbances were obtained by manipulating the amount of liquid component in the cement mass and thus changing the ratio of liquid component to powder. Although deviations in mixture proportions due to human operator error are usually in the range of a few to several wt%, for the purpose of statistical analysis, extreme imbalance scenarios were included. While these are, arguably, unlikely in clinical practice, the results obtained provided extended data for modelling trends over a wider range of deviations. The accepted range of deviations from the recommended ratio was −30% to +40% with a variable step of 10–15% [34]. Samples were prepared under room conditions, at 23 °C. For each cement, more samples were made than required by the above-mentioned standard, i.e., about 10 samples for each series, with the following inaccuracies (%*w*/*w*) of the liquid part: −30%, −15%, 0%, 10%, 20%, 40%. The cements were mixed manually, and the suitability of the mixture for moulding was assessed on the basis of the standard and the manufacturer’s recommendations, i.e., by observing whether fibres form between the cement and the glove when the finger leaves the surface, and then by repeating the test every 15 s the moment when the gloved finger first separates from the cement was recorded. A specially prepared mould was used to make these samples. After 24 h at room temperature, the finished samples were polished with an abrasive tool until the desired length was achieved. A visual assessment of the quality of the samples was carried out, and those with visible defects in the structure were rejected. The chemical composition of the two cements analysed is identical to a certain extent, i.e., in the composition of the bulk and liquid parts some components are the same. The main differences are in the type of radiopaque agent, the use of colourant and the antibiotic.

**Powder part:**

Shared components:

- polymethyl methacrylate (PMMA) and benzoyl peroxide (as initiator),
- radiopaque agent: barium sulphate (in CMW3), zirconium dioxide (in Palamed);

Additionally:

- antibiotic: gentamicin sulphate (in CMW3),
- colourant: E141—chlorophyllin (in Palamed).

**Liquid part:**

Shared components:

- methyl methacrylate (MMA),
- N,N-dimethyl-ptoluidine (DMPT) (as accelerator),
- hydroquinone (as stabiliser),

Additionally:

- colorant: E141—chlorophyllin (in Palamed, as colorant).

**Figure 2:**
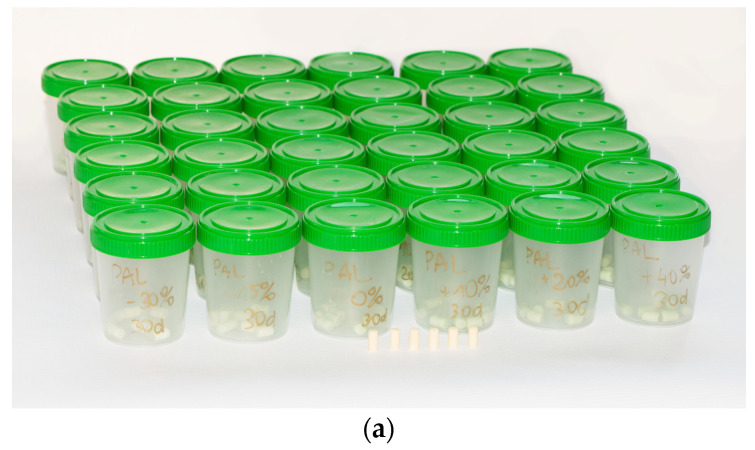

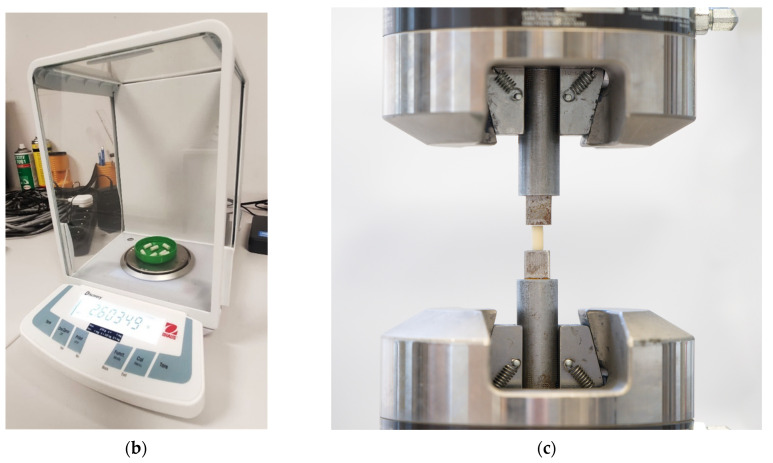
(**a**) Seasoned samples—PALAMED series, (**b**) weighing of seasoned samples and (**c**) specimen ready to be compressed, fixed in holders.

Figure 2a shows a group of samples prepared for seasoning. The samples were placed in separate containers in groups according to seasoning time: unseasoned, seasoned for 1 day, 10 days, 20 days and 30 days.

The samples were seasoned in Ringer’s solution, and their initial and final masses were monitored to determine the absorbency of the fluid (%*w*/*w*) at successive seasoning days, i.e., 1 day, 10 days, 20 days, 30 days. Before measurement, the samples were gently dried from excess fluid with a paper towel. The specimens were weighed just before the strength tests using Ohaus Discovery DV215CDM 210 g × 0.01/0.1 mg laboratory balance with a glass draftshield doors (Figure 2b).

### 2.2. Mechanical Testing

The individual series were tested using an MTS Bionix-Servohydraulic Test System (Eden Prairie, MN, USA) Figure 2c. The specimens were placed in the testing machine without any type of spacer between the cylinder and the machine plate. During the compressive loading, the machine plate was moved at a constant speed of 20 mm/min, and the resistance force [N] and deformation [mm] of the cement specimen were recorded. The test was carried out until the upper yield point was reached. Its value divided by the cross-sectional area of the sample was expressed as the compressive strength in MPa. In addition, the compression modulus, which determines the elasticity of the material, i.e., the slope of the stress/strain curve in the region of 2% elastic deformation, was measured. The tests were carried out at a room temperature of 23 °C.

### 2.3. Statistical Analysis

The obtained results were analysed statistically using TIBCO Statistica 13.3 software. The statistical significance of differences between individual groups of results, i.e., between strength and strength modulus of samples with a same composition and seasoned in different time, and between samples seasoned in the same time and made with different proportions of components was tested. A typical level of significance was assumed for the analyses, α = 0.05. In order to determine whether the analysed groups of results were characterised by a normal distribution, two tests were performed: W Shapiro–Wilk and K-S with Lillefors correction. The post-hoc multiple equation test was used to group the means and to separate homogeneous groups of statistically insignificant differences. Several such tests are available in the software used (e.g., Fisher’s test, Bonferroni’s test, Scheffé’s test and Tukey’s test, or tests for differences: Newman–Keuls and Duncan). They differ in the statistical method of performing analyses. In our study, the Tukey test was used in a variant for different numbers of compared samples, because not in every series the numbers of samples were identical.

## 3. Results and Discussion

### 3.1. Moisture Uptake

**Figure 3:**
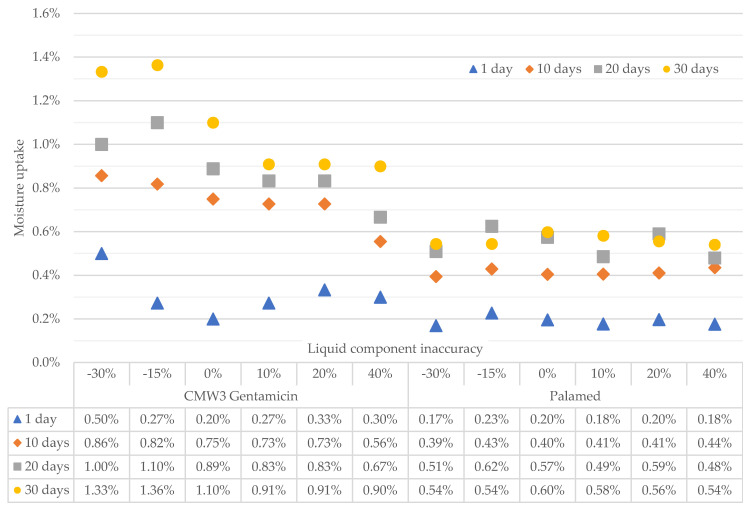
Results of the analysis of the absorption of Ringer’s solution by samples of different compositions during seasoning.

The change in weight of the seasoned specimens resulting from the uptake of Ringer’s solution was investigated prior to the strength tests. The results of the measurements were referred to the weight of the specimens before being placed in the solution and are presented in %*w*/*w* in Figure 3. 

A noticeable weight gain was observed during seasoning in Ringer’s solution of the CMW 3 Gentamicin cement. Importantly, this increment is greater for cements made with a deficiency of the liquid component than for cements with an excess of the liquid component. Palamed also shows absorption of Ringer’s solution, but on a much smaller scale. In addition, the weight gain does not seem to depend significantly on the accuracy of the liquid component of the cement, which shows that the cement appears therefore more resistant to liquid, at least in the analysed time range. This fact may be related to other material characteristics of the cement.

It can be seen that the fit of the linear model to the moisture uptake results (Table 1) obtained for the examined bone cements is very good in both cases (0.77 ÷ 0.98), which allows a conclusion to be made that such a model is correctly selected as a description of the time-course absorption processes of the Ringer’s solution. The effect of the inaccuracy of the liquid component on the absorption is clearly visible for CMW3 Gentamicin cement, where the parameter m decreases as the amount of the liquid component in the cement increases, i.e., cement with an excess of the liquid component absorbs the Ringer solution less well. In the case of Palamed cement—the stability of the absorption can be seen, i.e., irrespective of the cement composition studied, the slope of the linear trend line is fairly similar and the differences between the absorption of cements of different compositions analysed are minimal.

### 3.2. Compressive Strength 

**Figure 4:**
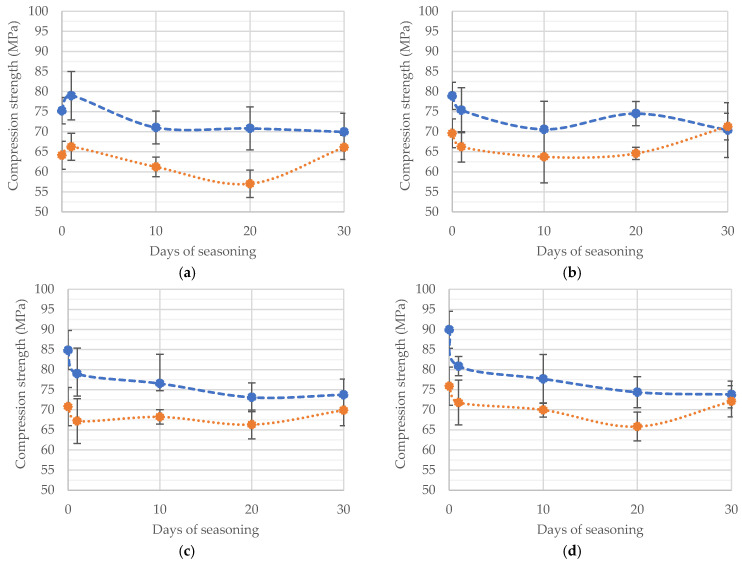

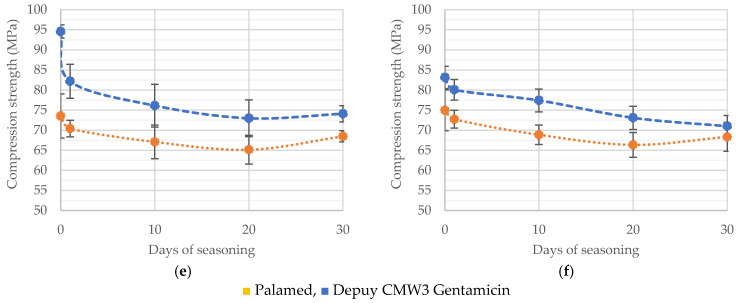
Change in average compressive strength of the cements tested as a function of their composition: (**a**) −30% liquid part (l.p.), (**b**) −15% l.p., (**c**) correct ratio, (**d**) +10% l.p., (**e**) +20% l.p., (**f**) +40% l.p.

Compressive strength tests were conducted immediately after weighing the seasoned specimens. According to the test plan and the previously mentioned ISO standard, the results obtained were averaged and, taking into account the standard deviation, are presented in the following diagrams. Figure 4 shows the compression strength results grouped according to the inaccuracy of the liquid component dosage measured with increasing seasoning time. The data clearly show that the average compressive strength decreases with seasoning time. This is particularly evident for cements made with an excess of the liquid component.

**Figure 5:**
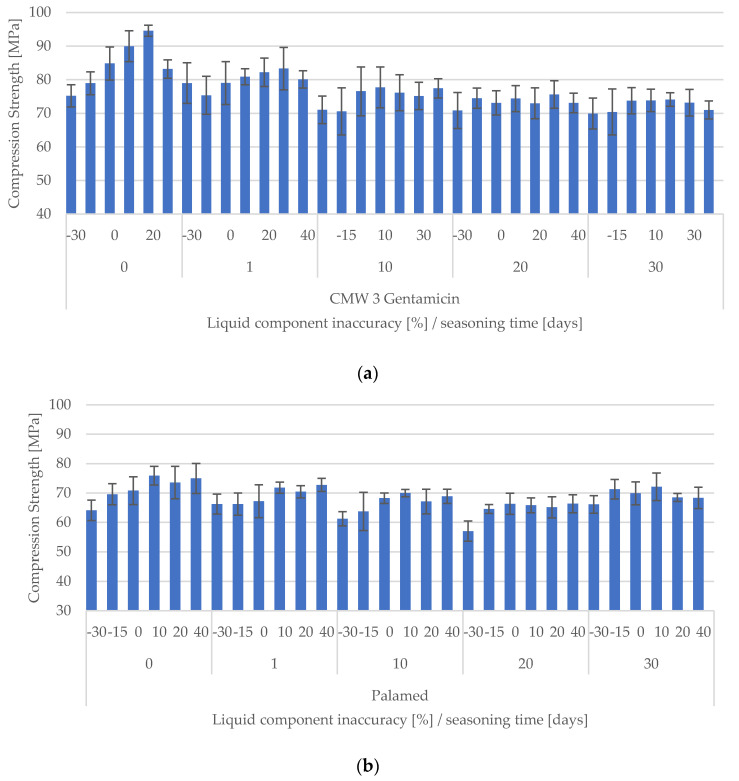
Summary results of compressive strengths of tested cements in relation to composition (**a**) DePuy CMW3 Gentamicin and (**b**) Palamed.

A summary of all the results obtained from the tests is shown in the comparison of manufacturing inaccuracy from seasoning time in Figure 5a,b.

A very first analysis of the results obtained reveals some regularities. For both cements, before seasoning, a deficiency of the liquid component (MMA monomer, accelerator and stabiliser) leads to a weakening of the compressive strength of the crosslinked cement. A slight excess (10–20%) strengthens the cement, while above this range the effect is again negative. Similar behaviour was recorded for seasoned cements, but the strength changes were not as large as for unseasoned cement. It seems that the longer both cements were seasoned, the less significant the effect of cement inaccuracy was, although large deficiencies (–30%) still resulted in a deterioration of compressive strength.

Statistical analyses were performed to show whether there were statistically significant differences between individual series of compression strength test results. The results of the Tukey post-hoc test, resulting in defining homogeneous groups of values that do not differ in a statistically significant degree, according to the adopted coefficient α = 0.05, are presented in Table 2. This allows to indicate the trends of real changes in strength properties of the cements tested. The * symbol indicates that particular groups of cements belong to homogeneous groups (1/2/3) of results that do not differ statistically on the assumed significance level. Only a careful discussion of the results of statistical analysis gives an answer to the question of real differences between cements after the assumed seasoning periods. Interestingly, despite clearly different average strength values, differences after seasoning were confirmed mainly for samples with an increased amount of liquid part. This may be due to the fact that cements with a reduced amount of liquid monomer gave cements with a larger spread of strength results, cements that were less uniform in their structure. These larger spreads made it impossible to confirm the existence of statistical differences between the groups.

**Figure 6:**
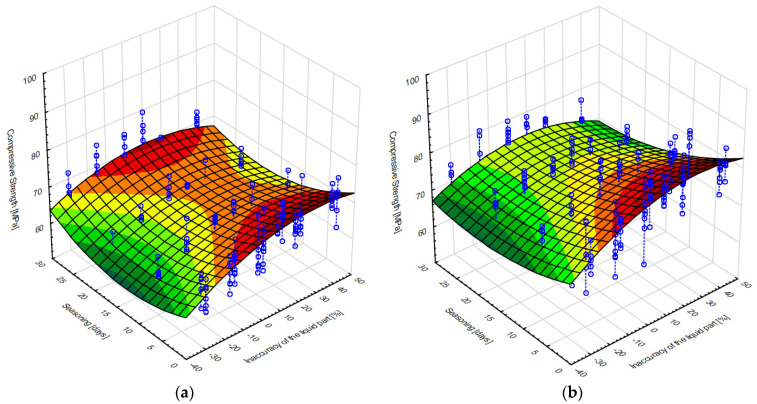
Surface model of compressive strength from liquid component inaccuracy and seasoning time for: (**a**) Palamed, (**b**) CMW3 Gentamicin.

Figure 6 shows the results of the mathematical modelling of the compression strength of the cements analysed—fitting a model of the form (1):

*Compressive strength* = *b*_1_ + *b*_2_ × *v*_1_ + *b*_3_ × *v*_2_ + *b*_4_ × *v*_1_^2^ + *b*_5_ × *v*_1_ × *v*_2_ + *b*_6_ × *v*_2_^2^ (1)

where:

- *b*_1_—free expression,
- *b*_2_*–b*_6_—coefficients at individual components,
- *v*_1_—liquid component inaccuracy (%),
- *v*_2_—seasoning time (days).

Table 3 summarises the parameters of the mathematical models shown in the figures above. As can be seen, apart from the free expression b_1_, the other coefficients of the model describing the dependence of strength on ratio inaccuracy and seasoning time, as well as their products and squares, are similar for both cements. The greatest difference is precisely in the free expression describing the strength when not taking into account the parameters analysed (inaccuracy and seasoning). According to the model obtained, Palamed is 10.65 MPa less compressive strength under these conditions, which is 13% less in relation to CMW3.

### 3.3. Compressive Modulus of Elasticity

**Figure 7:**
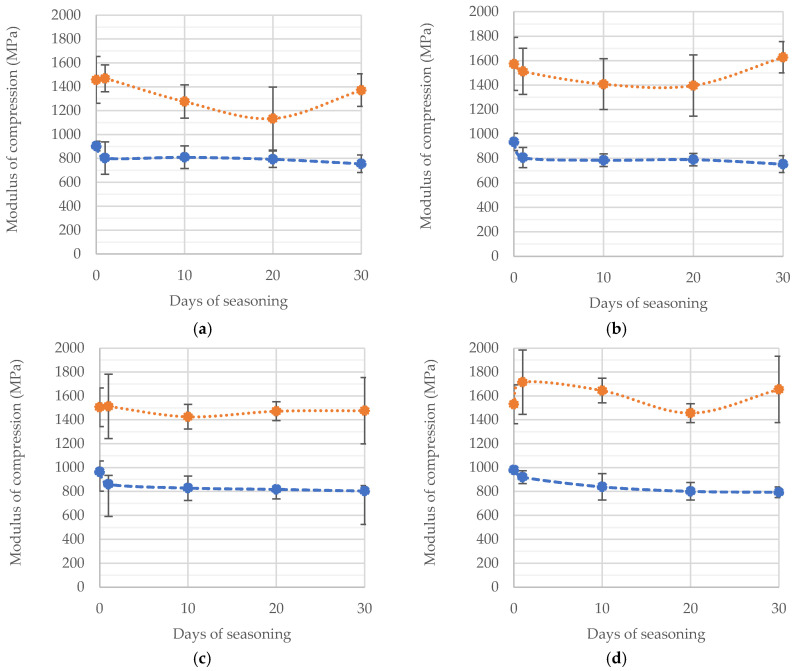

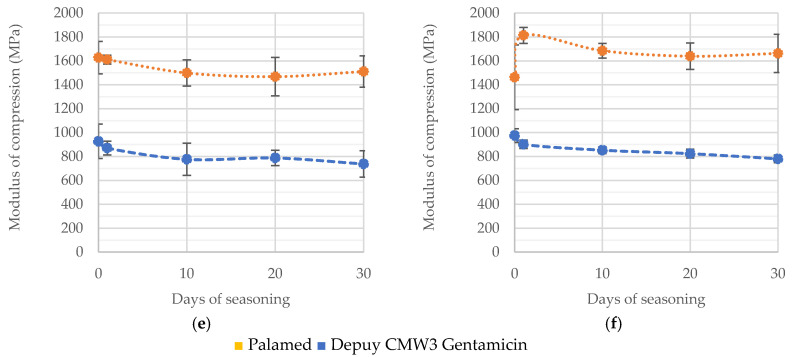
Variation of the average compressive modulus of the tested cements as a function of composition, i.e., (**a**) at −30% liquid part (l.p.), (**b**) −15%, (**c**) correct ratio, (**d**) +10% l.p., (**e**) +20% l.p., (**f**) +40% l.p.

Figure 7 shows the average values obtained experimentally for the modulus of longitudinal elasticity in compression of seasoned samples with various degrees of accuracy of proportions of the components. The differences between the tested cements can be seen at first glance. Palamed usually achieves 40 to 80% higher modulus values, which clearly shows how much more elastic the material is compared to CMW3. The variation of the average value of modulus depending on the amount of deficiency or excess of the liquid component in the cement mass, taking into account the seasoning time itself, does not seem to be very significant. The slight differences observed are unlikely to be statistically significant, mainly due to the standard deviation of the recorded series of results. This will be the subject of further analysis. However, it should be remembered that the stiffness or flexibility of a polymeric material is related to the length of the polymer chains, i.e., the result of the crosslinking process. Shorter polymer chains, on the other hand, may be related to the presence of unreacted resin molecules in the cement.

**Figure 8:**
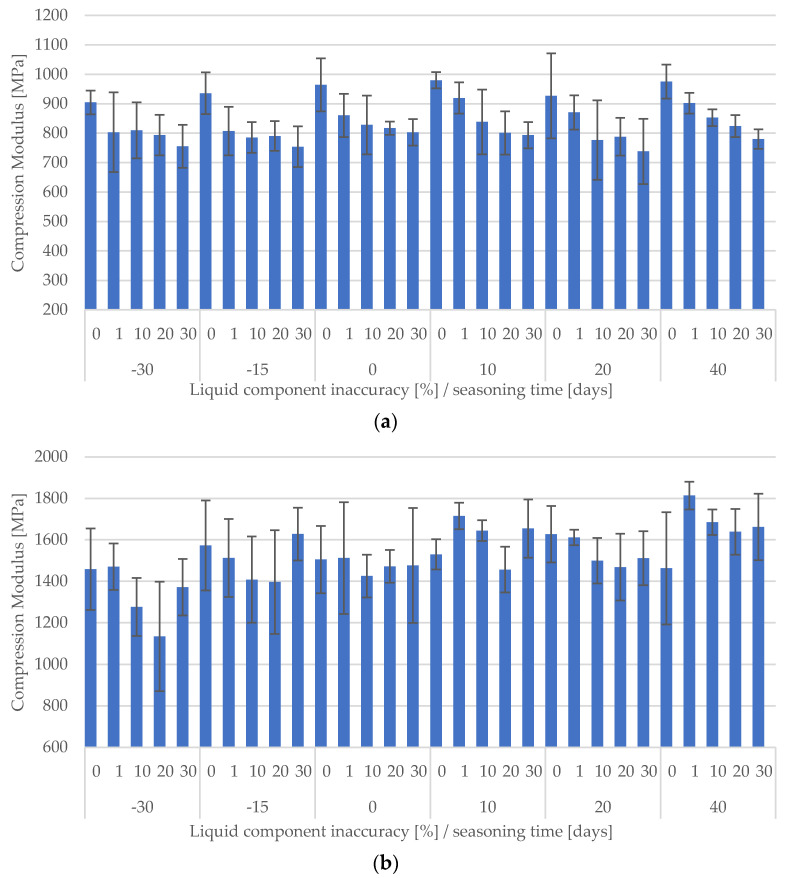
Summary results of the compressive modulus of the tested cements depending on the seasoning time (**a**) CMW 3 Gantamicin, (**b**) Palamed.

A summary of all test results obtained is presented as a dependence of manufacturing inaccuracy on seasoning time in Figure 8a,b.

A preliminary analysis of the results obtained allows us to note that in the case of DePuy CMW3 Gentamicin cement (a), with increasing time spent in Ringer’s solution, the average values of the compressive modulus decreased significantly, most notably just after the beginning of seasoning, i.e., after soaking in the solution. This shows the importance of simulating near-real conditions for this type of testing. This behaviour of the cement was observed basically regardless of how precisely the recommendations for the ratio of liquid and powder components were followed. Palamed reacted with a change in compressive modulus as a result of seasoning in a somewhat less obvious manner, i.e., the rather large variation in the results obtained within individual batches makes it difficult to observe any apparent regularities and draw general conclusions based on them. This is interesting because the same series tested for compressive strength gave clearer results and changes in strength depending on the degree of inaccuracy of the cement or seasoning time were clearly observable.

The results of the statistical analysis, showing whether there are statistically significant differences between individual series of test results of the compressive modulus, are presented in Table 4. Again, the Tukey post-hoc test was used, and similarly to the statistical analysis of compressive strength, here also the result of the analysis was the grouping of individual series of results into homogeneous groups, which do not differ from each other to a statistically significant degree, according to the assumed coefficient α = 0.05. This allows for distinguishing the trends of real changes of the compressive modulus of the studied cements.

**Figure 9:**
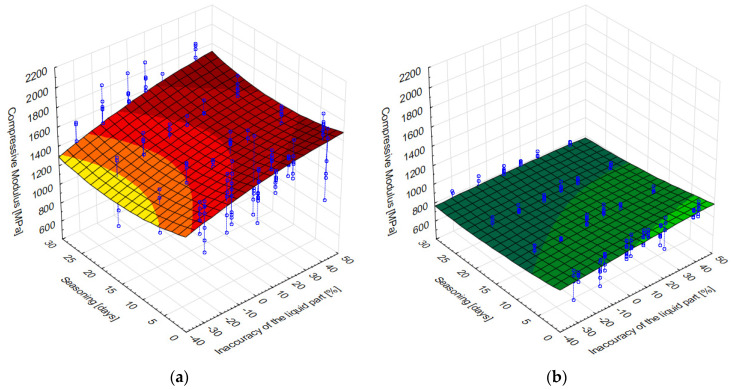
Surface model of the compression modulus from liquid component inaccuracy and seasoning time for: (**a**) Palamed, (**b**) CMW3 Gentamicin.

Figure 9 shows the results of the mathematical modelling of the compression modulus of the cements analysed—matching a model identical to (1).

Table 5 summarises the parameters of the mathematical models shown in the above figures. As can be seen, apart from the free expression *b*_1_, the other coefficients of the model describing the dependence of the modulus on the ratio inaccuracy and seasoning time as well as their products and squares, are of a similar nature (increasing or decreasing the modulus). However, they differ in magnitude. The most important value—*b*_1_ is approx. ¾ larger for Palamed than for CMW3, as observed earlier.

## 4. Discussion

An important disadvantage of polymeric biomaterials is their susceptibility to degradation and the related loss of original properties with the time spent in aggressive environmental conditions of the human body. The cement is exposed to the aggressive body fluid environment throughout the implantation time [70]. On the other hand, resistance to this environment is crucial for the long-term performance of cements, i.e., the long-term stability of the implanted prosthesis, e.g., the hip joint replaced by endoprosthesis, as it prevents potential joint damage, need revision procedures, or is a source of discomfort for the patient and financial burden. It has also been shown that the mere fact of using fluids to rinse the bone canal at the stage of implantation will affect the polymerisation process of the cement and thus its mechanical properties after curing [71]. Cement composition imbalance can lead, as shown in this paper using Palamed cement as an example, to a reduction in compressive strength below the 70 MPa value required by the ISO standard [69], which occurred when the liquid component was insufficient. As shown in the study described in this paper, over the observed 30-day period, in an environment simulating in vivo conditions, the strength properties of the cement will also decrease below abovementioned border value.

Moreover, release of MMA during prostheses implantation can lead to bone cement implantation syndrome (BCIS), which can result in hypotension, hypoxia and lung emboli formation [72]. It is shown that BCIS is associated with an increased risk of 30-day mortality following joint replacement [73]. The exact aetiology of BCIS is unclear, however factors such as inflammatory, thermic and complement activation have been proposed [72,74]. Released during implantation PMMA has also negative effect on lungs, by contributing to the formation of emboli which originates from PMMA [75]. Given the above, it shows that exact understanding of the mechanisms responsible for altering cement components has great impact not only on survivor rate of TJA, but also on mortality during surgery. In the human body, bone cement is subjected to very hostile conditions which induce surface changes in the bone cement [76], which can cause micromotion at the interference site due to reduction of its strength. This phenomenon is suggested to be a major cause of aseptic loosening of TJR [77,78].

Another critical factor determining the strength of a cement is its composition, especially any modifications. Many studies have been conducted in which modification of the cement composition by the addition of various materials could potentially improve the characteristics of the finished, hardened cement (e.g., [50,79,80,81,82]). The starting point of the study described in this paper was the modification of the cement not by changing its composition but by changing the proportions of the liquid monomer and polymer powder (PMMA). Such imprecision, which may occur in surgical practice, has the character of an accidental error and may result in a change in the characteristics of the cement itself and, above all, in the prosthesis-cement-bone canal joint. The occurrence of disturbances in the recommended proportion of components forming polymeric materials has been studied, among others, in the problem that is functionally similar to the issue of bone cements, i.e., in the case of polymeric adhesive materials (e.g., epoxy [83,84,85,86]). As in the case of bone cements, epoxy adhesives prepared in a different ratio than the recommended may produce a different degree of crosslinking of the material, which will ultimately affect its strength and the strength of the bond that is produced with it. Deviations from the recommended proportions will lead to the production of a more or less brittle material with better parameters of resistance to stress, temperature, or the work of aggressive agents such as water. By precisely defining the correct composition (with a precise value or range of values), the manufacturer guarantees the best possible performance parameters of the material, i.e., not only the mechanical strength properties, but a range of other properties, comprehensively and optimally. In the case of cements, a change in chemical composition (in terms of proportions of components) may also be accompanied by certain adverse effects related to the fact that such a cement works inside the human body. Therefore, any purposeful modification of the proportions of components used to make a cement must take into account these other features of the material, not covered by the research presented in this paper. For example, if some unreacted liquid part remains in the cement mass, it may lead to allergies, irritations and even more serious complications. Unreacted loose monomer, on the other hand, may leave the cement and enter the bloodstream, which will have a negative effect on the whole body. Such a reaction is not likely to be mutagenic or carcinogenic, but certainly allergenic [87,88,89].

The research undertaken in this study provides answers only to questions regarding mechanical changes in the cement material. Analysed results of elasticity modulus tests allow observation of small influence of cement inaccuracy on modulus values in case of CMW3, i.e., neither liquid part deficiency resulting in monomer powder excess, nor the reverse variant significantly changed the cement stiffness. Palamed showed a statistically significant increase in modulus in several variants of seasoning time, usually with an increase in the amount of liquid part in the cement composition. The modulus of elasticity largely depends on the degree of crosslinking of the polymeric structure [90]. Long chain material will have higher Young’s modulus values—greater stiffness. However, this is not always desirable. Cement that has lower strength but is more flexible will allow the joint to work longer, particularly in the case of non-uniform loading, due to a more uniform distribution of stress. On the other hand, one of the most important characteristics of cement, compressive strength, decreases significantly in the case of CMW3 cement made in the correct composition after 20 days of seasoning. In the case of an excess of the liquid component used to make the cement, a significant decrease in strength was recorded already after 1 day of seasoning. The results obtained for Palamed were not completely conclusive in the whole examined range. In spite of lower mean values of compressive strength of samples after 10–20 days of seasoning, they were not always statistically significant. Similarly, both cements reacted to changing the amount of liquid component in the composition. Typically, the more liquid component the higher the average strength values, but this was not always statistically significant.

One reason for the observed changes may be the change in the degree of porosity of the final cement, which plays a major role in total implant failures of, for example, the hip joint, as it is directly proportional to loosening and instability. A reduction in the number of pores results in a stronger fixation of the prosthesis. Models exist to estimate porosity at the cement-prosthesis interface [91]. Research is also known to develop methods to prevent excessive harmful porosity in cements [92,93]. In the case of the results reported in this work, porosity may be one of the factors leading to the observed changes in the absorbency of Ringer’s solution by the cements studied. The study showed significant differences between the absorbency depending on how the cement was made. Depuy CMW3 Gentamicin was able to absorb larger amounts of liquid, especially when the cement was made with a deficiency of the liquid component of the cement. This may be related to the lower degree of polymerisation of the cement and the remaining unreacted part—the powder—which absorbs fluid to a greater extent than fully crosslinked cement.

### Limitations of the Study and General Future Directions 

The choice of conditions adopted in this study is an attempt to simulate the human body conditions after the process of implantation of a prosthesis using bone cement. Therefore, it might seem that they reproduce quite well the influence of changes of the analysed factors on the strength of the bone cement. However, due to its nature, the study has certain limitations. First of all, cements in real conditions are subjected to cyclic loading, which also influences the behaviour of the material, while in this study, for simplicity, the focus was on static compression. Moreover, a relatively short seasoning period covering the initial month after implantation of the cemented prosthesis was adopted. The study could therefore be extended to include a longer time horizon, e.g., monthly intervals between testing specimens for a total duration of at least one year of testing. This allowed testing the long-term effect of the factors analysed in this study. Moreover, the form of seasoning assumed in the experimental design, i.e., at room temperature, may also be important in terms of relating the presented results to real conditions. For this reason, similar studies, but at 36.6 °C seem highly important. The authors are aware of the fact that the range of inaccuracy of the cement composition adopted in this study does not fully correspond to the possibility of random occurrence, although an exaggerated extension of this range allows to more clearly indicate the direction of changes in the cement properties in response to a change in this parameter, which due to the inaccuracy of measurements (standard deviation) of the obtained results could be difficult or even impossible to demonstrate.

## 5. Conclusions

As shown in the study, seasoning the tested cements in Ringer’s solution, which was used to simulate real-life in vivo conditions, significantly affects the strength of bone cements. This is related, among other things, to the absorption of the cement fluid. However, the longitudinal compressive modulus, which determines the elasticity of the material, turns out to be a more seasoning-resistant parameter. Limited resistance of cements to inaccuracy of workmanship related to incorrect ratio of liquid to powder was also demonstrated and the mechanisms of changes in the cement structure occurring due to these changes were discussed.

## Figures and Tables

**Table 1:** Parameters of linear model of moisture uptake (mx + b).

Inaccuracy of Components Ratio (Liquid Part)	Palamed			CMW3		
	m	b	R^2^	m	b	R^2^
−30%	0.0035	0.0025	0.8853	0.0081	0.0063	0.9724
−15%	0.0033	0.0046	0.7770	0.0088	0.0013	0.9365
0%	0.0036	0.0028	0.8993	0.0065	0.0031	0.7926
+20%	0.0035	0.0026	0.9259	0.0051	0.0059	0.8775
+40%	0.0034	0.0035	0.8520	0.0057	0.0037	0.9819
+50%	0.0031	0.0034	0.8338	0.0049	0.0053	0.9746

**Table 2:** Results of statistical analysis of compressive strength test results (*—samples within homogeneous groups).

Liquid Part Inaccuracy	Seasoning [Days]	CMW 3 Gentamicin				Palamed			
		Mean Strength [MPa]	1	2	3	Mean Strength [MPa]	1	2	3
−30%	0	75.21	*			64.12	*		
	1	76.30	*			66.23	*		
	10	71.03	*			61.24	*	*	
	20	70.81	*			57.02		*	
	30	69.94	*			66.10	*		
−15%	0	78.92	*			69.58	*	*	
	1	75.36	*			66.22	*	*	
	10	70.57	*			63.73	*		
	20	74.49	*			64.58	*	*	
	30	70.38	*			71.28		*	
0%	0	84.81		*		70.79	*		
	1	78.98	*	*		67.20	*		
	10	76.53	*	*		68.22	*		
	20	73.10	*			66.31	*		
	30	73.73	*			69.89	*		
10%	0	89.94			*	75.87		*	
	1	80.86		*		71.81	*	*	
	10	77.70	*	*		68.97	*		*
	20	74.38	*			65.82			*
	30	73.82	*			72.10	*	*	
20%	0	94.57			*	73.53		*	
	1	82.18		*		69.68	*	*	
	10	76.12	*	*		67.09	*		
	20	72.97	*			65.14	*		
	30	74.09	*			68.48	*	*	
40%	0	83.15			*	73.17	*		
	1	80.08		*	*	72.73	*		
	10	77.42		*		68.85	*	*	
	20	73.09	*			66.34		*	
	30	71.00	*			68.34	*	*	

**Table 3:** Compressive strength model fitting parameters.

	*b*_1_	*b*_2_	*b*_3_	*b*_4_	*b*_5_	*b*_6_
Palamed	71.3464	0.1506	−0.7459	−0.0025	−0.0029	0.0236
CMW3 G	82.0006	0.1541	−0.722	−0.0029	−0.0035	0.0148

**Table 4:** Results of statistical analysis of compression modulus test results (*—samples within homogeneous groups).

Liquid Part Inaccuracy	Seasoning [Days]	CMW 3 Gentamicin					Palamed			
		Mean Modulus [MPa]	1	2	3	4	Mean Modulus [MPa]	1	2	3
−30%	0	904.48	*				1457.99	*		
	1	806.33	*				1470.34	*		
	10	809.93	*				1276.22	*	*	
	20	793.59	*				1134.26		*	
	30	755.62	*				1371.70	*	*	
−15%	0	935.71		*			1572.86	*		
	1	806.98	*				1512.53	*		
	10	785.34	*				1407.61	*		
	20	790.46	*				1396.41	*		
	30	753.97	*				1627.89	*		
0%	0	964.26		*			1504.81	*		
	1	860.35	*	*			1512.06	*		
	10	828.00	*				1425.05	*		
	20	816.98	*				1471.68	*		
	30	803.12	*				1476.22	*		
10%	0	979.96			*		1529.62	*	*	
	1	919.66		*	*		1714.86			*
	10	838.39	*	*			1636.84	*		*
	20	800.83	*				1456.18		*	
	30	793.51	*				1654.31	*		*
20%	0	926.79	*				1627.03	*		
	1	870.62	*				1592.06	*		
	10	776.62	*				1499.11	*		
	20	788.06	*				1468.36	*		
	30	738.10	*				1511.07	*		
40%	0	975.34				*	1452.31	*		
	1	901.90			*		1813.58		*	
	10	852.64		*	*		1684.67	*	*	
	20	823.97	*	*			1638.91	*	*	
	30	780.30	*				1662.44	*	*	

**Table 5:** Fitting parameters of the compression modulus models.

	*b*_1_	*b*_2_	*b*_3_	*b*_4_	*b*_5_	*b*_6_
Palamed	1570.73	3.564	−15.5502	−0.0263	0.048	0.4738
CMW3 G	897.64	1.1818	−8.7537	−0.0061	−0.0333	0.1616

## Data Availability

Data presented in this study is available from corresponding authors upon request.
